# An Epidemiological Update on Indoor Tanning and the Risk of Skin Cancers

**DOI:** 10.3390/curroncol29110699

**Published:** 2022-11-17

**Authors:** Clio Dessinioti, Alexander J. Stratigos

**Affiliations:** Melanoma and Skin Cancer Unit, 1st Department of Dermatology-Venereology, National and Kapodistrian University of Athens, Andreas Sygros Hospital, 16121 Athens, Greece

**Keywords:** indoor tanning, sunbeds, solarium, skin cancer, melanoma, basal cell carcinoma, squamous cell carcinoma, prevention

## Abstract

Indoor tanning (sunbeds, solarium) uses artificial ultraviolet radiation (UVR) to stimulate cosmetic tanning of the skin. Indoor tanning has been officially classified as a human carcinogen in 2009 by the International Agency for Research on Cancer of the World Health Organization (WHO). The differences in the prevalence of sunbed use across countries and over the years highlight underlying legislative, climatic, and cultural differences. Indoor tanning-seeking behaviors may be driven by motivations for an appealing appearance, largely influenced by gender and age, and several misconceptions that a prevacation tan safeguards the skin, that sunbeds can be used to treat acne or to increase vitamin D, or that tanning is a healthy habit. This review provides an epidemiological update on the prevalence of sunbed use, who tends to use sunbeds and why, and details the current evidence on the association of sunbeds with skin cancers, including cutaneous melanoma, basal cell carcinoma (BCC), and cutaneous squamous cell carcinoma (cSCC). A statistically significant higher risk of cutaneous melanoma, BCC and cSCC with the use of sunbeds has been consistently demonstrated. This risk of skin cancer is even higher with the more frequent use of sunbeds, underscoring a dose–response relationship, and in those first exposed to sunbeds at a younger age. Preventive measures against sunbed use include legislation restricting sunbed use, educational campaigns to inform and discourage from indoor tanning, as well as using the internet, online advertising messages and the social media to reach larger audiences and to promote an untanned appearance.

## 1. Introduction

Ultraviolet radiation (UVR), consisting predominantly of UVA (320–400 nm) and a small fraction of UVB (280–320 nm) wavelengths, is emitted by the sunlight and sunbed indoor tanning devices, and is responsible for tanning (natural or artificial tanning, respectively) [1]. In modern times, tanning (from the sun or sunbeds) presents as a wolf in sheep’s clothing. On the one hand, part of the public embraces tanning and perceives it as fashionable due to advertised and long-standing social constructs of beauty. On the other hand, tanning is a priori a marker that the skin has been under sun (UVR) “attack”.

Tanning represents acquired skin pigmentation that is induced after exposure to UVR, in contrast to the constitutional pigmentation that characterizes baseline skin color. While constitutional skin pigmentation is influenced by a variety of factors, including melanin, capillary blood flow, cutaneous chromophores (lycopene, carotene), and dermal collagen, it is the production of melanin by the melanocytes that primarily determines skin color [2]. Melanin is produced in cutaneous melanocytes, via the biochemical pathway of tyrosine, either as the brown-black eumelanin or as the yellow-red pheomelanin which differ in color and in the size, shape and packaging of their granules [2,3]. Pheomelanin is more photolabile and can produce hydrogen peroxide, superoxide and hydroxyl radicals, that result in oxidative stress and further DNA damage [3]. The pigmentation signaling pathway is under a complex genetic control and the ratio of eumelanin to pheomelanin determines skin color [2,4,5]. Tanning occurs after the UVR-induced production of melanin is packaged and delivered to keratinocytes by melanosomes. The melanosomes resemble umbrellas and protect the sun-exposed side of nuclei of the keratinocytes, after exposure to UVR [3].

However, tanning itself may be triggered following DNA damage from UVR exposure [3]. UVA-induced DNA damage occurs mostly indirectly, through the production of melanin, that in turn stimulates the production of reactive oxygen species (ROS) which induce single-stranded breaks in DNA, in key genes implicated in skin carcinogenesis. UVB induces direct DNA damage through the creation of photoproducts, such as cyclobutane pyrimidine dimers (CPDs), that are mutagenic if left unrepaired. However, CPDs seem to also play a central role in UVA-induced mutagenesis, as UVA may induce CPDs as a major pro-mutagenic DNA photoproduct [6]. In addition, UVA and UVB, even at suberythematogenic doses, may mediate skin carcinogenesis through immunosuppressive pathways, after cell membrane damage, DNA damage, and trans-urocanic isomerization [1,7,8,9].

Indoor ultraviolet radiation tanning (referred as indoor tanning from hereon) uses artificial UVR to stimulate cosmetic tanning of the skin [10]. The International Agency for Research on Cancer (IARC) regards the whole spectrum of UVR as well as UV-emitting tanning devices as first-group carcinogens alongside tobacco smoking and asbestos [11]. Regardless of the well documented harmful effects of indoor tanning, there is still a considerable frequency of sunbed use which poses questions on the motivations for tanning-seeking behaviors and whether the public is aware of the health harms and carcinogenicity induced by sunbeds, including the significant risk of skin cancers.

This is a scholarly review, and not a systematic review, aiming to present a comprehensive synthesis of the prevalence of sunbed use across various countries, which groups tend to use sunbed use and why, and summarize the up-to-date evidence on the association of sunbeds with skin cancers, including cutaneous melanoma, basal cell carcinoma (BCC), and cutaneous squamous cell carcinoma (cSCC). With this aim, we performed searches in PubMed using the terms related to each topic addressed, e.g., “indoor tanning”, “sunbed” and “melanoma”, “skin cancer”, “nonmelanoma skin cancer”, “basal cell carcinoma”, and “cutaneous squamous cell carcinoma”. Moreover, we carried out secondary referencing by manually reviewing reference lists of assessed articles. 

## 2. Epidemiology of Sunbed Use: Variation across Countries and among Individuals

**Figure 1:**
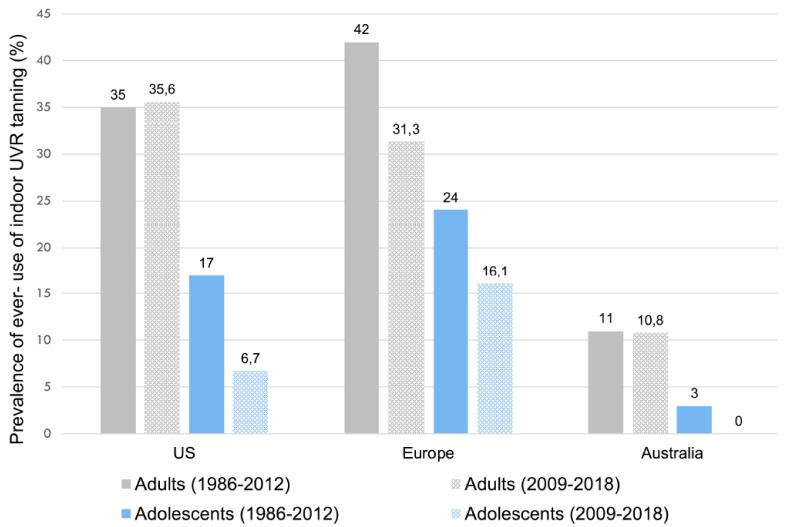
Summary prevalence estimates (%) reported in published meta-analyses on the ever-use of indoor UVR tanning. (The prevalence for the years 1986–2012 is reported in Wehner et al. [12], and for the years 2009–2018 in Rodriguez-Acevedo et al. [13]).

**Figure 2:**
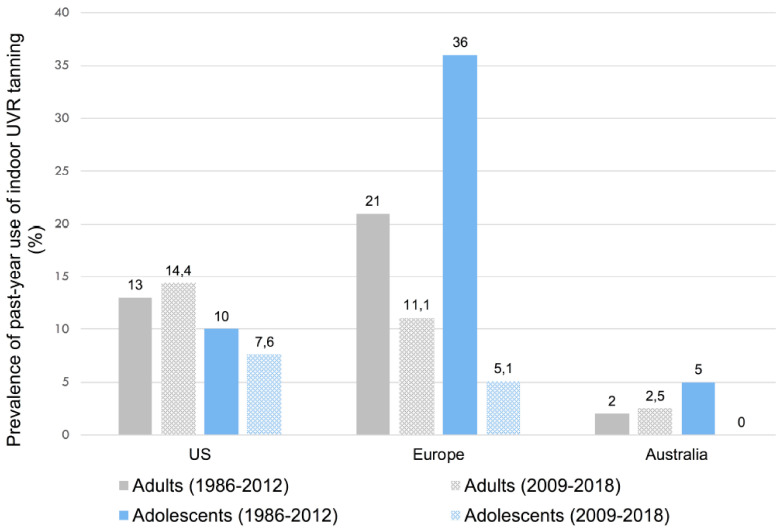
Summary prevalence estimates (%) reported in published meta-analyses on the past-year use of indoor UVR tanning. (The prevalence for the years 1986–2012 is reported in Wehner et al. [12], and for the years 2009–2018 in Rodriguez-Acevedo et al. [13]).

Despite the documented harmful effects of indoor tanning and restrictive legislation, a considerable prevalence of indoor tanning use has been reported across countries worldwide. The prevalence of indoor tanning use varies according to individual characteristics such as the age and gender, and according to the countries studied, (summarized in Table 1, Figure 1 and Figure 2). Australia shows the lowest prevalence of indoor tanning use. The meta-analysis of Wehner et al. (1986–2012) reported a prevalence of ever-use of indoor tanning in adults ranging from 11% in Australia to 35% in the US and 42% in Europe (Figure 1). The past-year prevalence of indoor tanning use was 18.3% in adolescents and 14% in adults, overall, varying across regions. Past-year use of indoor tanning in adults ranged from 2% in Australia, to 13% in the US, and to 21% in Europe (Figure 2) [12]. Rodriguez-Acevedo et al. performed a meta-analysis on the prevalence of indoor tanning, from data collected after the 2009 WHO classification of indoor tanning as a carcinogen (2009–2018) [13]. Since 2009, the worldwide prevalence of ever exposure to indoor tanning was 9.7% for adolescents and 33.4% for adults. The past-year prevalence was 6.7% in adolescents and 12.5% among adults. Past-year use of indoor tanning in adults ranged from 2.5% in Australia, to 11.1% in Europe, and to 14.4% in the US (Figure 2) [13]. Including the gender, the worldwide prevalence of past-year indoor tanning was higher in women compared to men (16.8% vs. 8.5%, respectively) and in adolescent girls compared to adolescent boys (8.9% vs. 3.9%, respectively), in the meta-analysis of Rotriguez-Acevedo et al. [13] (Table 1).

In the Euromelanoma survey study in 227,888 individuals from 30 European countries (2009–2014), the overall prevalence of ever-use of sunbed was 10.6%, with very large variations across countries. Belgium, Denmark, Estonia, Hungary, Italy, Latvia, and Spain had the higher prevalence of sunbed use of 18–27% (Table 1). The majority of sunbed users were light users (<20 sessions/year, <10 years). The highest proportion of heavy users (>20 sessions/year) was found for Turkey (60%), Malta (40%), Hungary (19.1%), Russia (19%), and Spain (17.1%). Indoor tanning was reported by 5.9% of adolescents (<20 years old), 17% of young adults (20–35 years old) and 8.3% of adults/elderly (>35 years old). Overall, sunbed use was more prevalent for females compared to males, independently from age, education, skin type and year of survey. Moreover, sunbed use was significantly more frequent by those with darker skin types in 14 countries, and by fairer skin types in Switzerland [14]. In another Euromelanoma report, regarding the prevalence of sunbed use according to age, young adults were found to be the most frequent sunbed users in European studies, and variable rates of sunbed use were reported in adolescents in Sweden, the UK, Italy, Germany, France, Denmark, and the Netherlands [15]. A recent study by the Italian Cancer League in 3692 participants in the skin cancer prevention campaign across 18 Italian centers reported a prevalence of sunbed use of 2.2% before the age of 15 years and 22.2% after the age of 15 [16]. 

The differences in the prevalence of sunbed use across countries highlight underlying climatic and cultural differences. Sunbed use has been associated with latitude [14,17]. A European case-control study (1999–2001) reported higher prevalence of indoor tanning in Northern Europe and lower prevalence in the South [17]. More recently, in the Italian Cancer League study (2018–2019), the prevalence of sunbed use was significantly higher among residents in Northern Italian regions (22%) compared to the Center (17%) and South of Italy (6%) [16]. On the other hand, the risk of melanoma with sunbed use did not differ with variations in latitude in the meta-analysis of Boniol et al. [18]. The Euromelanoma study (2009–2014) used the term ‘Baltic particularity’ to describe the high prevalence of sunbed use among young adults (<35 years old) that was reported in Estonia, Lithuania and Latvia. The authors suggested that this could be attributed to the consumerism and beautification methods incurred by the rapid globalization that occurred in these countries in recent years [14]. In addition, this study used the term ‘Scandinavian particularity’ to describe the high prevalence of sunbed use among adolescents in Norway, Belgium, Denmark, and Sweden [14], as previously described [19,20,21,22,23,24,25]. These findings underscore the importance of raising awareness on the sunbed-related risks and changing intentions and attitudes towards artificial tanning among adolescents in Scandinavian countries [14].

Over the years, the prevalence of past-year indoor tanning has decreased. Rodriguez-Acevedo et al., performed a systematic review and meta-analysis on the prevalence of indoor tanning, from data collected after the 2009 WHO classification of indoor tanning as a carcinogen (2009–2018) [13]. The change of the prevalence of indoor tanning over time was assessed by comparing with the results of a previous meta-analysis of Wehner et al. The prevalence of past-year indoor tanning among adolescents was 6.5% (95% CI: 3.3–10.6) for the period 2013–2018 [13], compared with 22% (95% CI: 17.2–26.8) reported for the 2007–2012 period by Wehner et al. [12]. This was reported as a statistically significant reduction of 70%. The indoor tanning prevalence among adults was 10.4% for 2013–2018 [13] compared with 18.2% for 2007–2012 [12], which was not a statistically significant change.

## 3. Motivations and Influences for Sunbed Use

A variety of motivations and influences are determinants of indoor tanning-seeking behaviors, including motivations for an appealing appearance, largely driven by gender and age, and several misconceptions that a prevacation tan safeguards the skin, that sunbeds can be used to treat acne or to increase vitamin D, or that tanning is a healthy habit.

Beautification motives of looking attractive were among the primary reasons for sunbed use in Europe, as was the influence of peers/parents using sunbeds [15]. The typical indoor tanner is female and late adolescent or young adult [16,26,27]. A review of determinants of sunbed use in Europe reported that the typical sunbed users in Europe are young-adult women, with a medium/high socio-economic status. In this study, women were 2–3 times more likely to use indoor tanning than males across all age groups [15]. A higher prevalence of sunbed use has been reported in sexual-minority men (including men who identify as homosexual, gay, bisexual, or other) compared to heterosexual men [28,29]. Motivations for indoor tanning among sexual-minority men include perceived appealing appearance, mood elevation, and perceived ‘healthy’ appearance of tanned skin [29,30]. 

Several misconceptions may motivate towards sunbed use. A common reason for sunbed use reported in European studies, was the wish for a ‘prevacation tan’. Most users believed that artificial tanning before the sun exposure during holidays would be beneficial for them, based on the false idea that it prepares the skin for further sun exposure [15]. However, inside the sunbeds, almost 100% of the body surface is exposed to UVA with higher amounts of irradiation compared to the partial body surface exposure to lower amounts of UVA during natural sunshine [31]. UVA irradiance emitted from indoor tanning devices has been measured to be much higher than from nature sun [10]. Moreover, as described above, tanning itself is a marker of UVR-induced DNA damage and there is no safe limit for exposure to UV radiation from sunbeds for cosmetic purposes.

Further misconceptions may promote the use of sunbeds for acne or vitamin D deficiency [15,32]. Patients with acne may expose themselves to indoor tanning due to the false perception that UVR is used to improve their acne. However, the use of UVR is contra-indicated for acne in the current European guidelines [33]. Devices emitting blue light may be used for the treatment of acne. However, blue light is part of the visible light spectrum, with a wavelength of 420 nm, and it has no carcinogenic effects. This is in sharp contrast with indoor tanning devices that emit the carcinogenic UVR spectrum. In these lines, sunbeds should not be used to increase vitamin D due their carcinogenic effects [15]. The false perception that tanning is healthy may arise from the presence of tanning beds in gyms and fitness facilities that are linked to exercise-associated health benefits and well-being [34,35]. In Canada, nearly half of the gyms offer indoor tanning, as do some of the largest American gym chains [34,36]. A survey in 636 indoor tanners reported that 24.2% had tanned at least once in a gym. Compared to other indoor tanners, individuals who had tanned in a gym were younger, more physically active, and more likely to be at risk for tanning dependence [34].

## 4. Sunbed Use and Risk of Cutaneous Melanoma

Several systematic reviews and meta-analyses have consistently established a statistically significant higher risk of cutaneous melanoma with the use of sunbeds (summarized in Table 2) [18,37,38,39,40,41,42]. Nevertheless, the quantification of the measure of the association of sunbed use with melanoma risk is hindered by limitations inherent to the design of individual studies. The most recent meta-analysis of An et al. (2021), included 36 studies in 14,583 melanoma cases and reported that the risk of cutaneous melanoma increased by 27% for those ever exposed to indoor tanning (RR: 1.27, 95% CI: 1.16–1.39) [37]. Similar risk estimates have been reported in previous meta-analyses (RR ranging from 1.15 to 1.27) (Table 2).

A dose-response relationship has been consistently shown between the amount of sunbed use and the risk of melanoma (Table 2). A significantly higher risk of melanoma was associated with 10 or more annual times of sunbed use [37,38,39] and with high [18] or longest exposure [42]. The meta-analysis of Boniol et al. (2012) reported a 1.8% increase in risk of melanoma for each additional session of indoor tanning per year [18].

The younger age at first exposure to sunbeds has been associated with significantly higher risk of melanoma in individual studies [44,45,46,47,48], systematic reviews and meta-analyses [18,37,41,49] (Table 2). The study of Lazovich et al., was the first to investigate age-specific and sex-specific associations between indoor tanning and melanoma and suggested that sunbed use may be contributing to the steeper increase in melanoma rates in younger women [44]. Age before 35 years old and ever sunbed use, as well as repeated/prolonged use between 10 and 39 years of age have been associated with significant increase in melanoma risk (75% and 237%, respectively) [18,41,50]. The meta-analysis by the IARC in 2007, showed a 1.75-fold higher risk of melanoma with the first use of tanning beds before the age of 35 [41]. In the meta-analysis of Boniol et al. (2012), there was a 1.59-fold higher risk of cutaneous melanoma for those that first used sunbeds at an age younger than 35 [18]. In the meta-analysis of An et al. (2021), exposure to indoor tanning before the age of 20 was associated with 1.47-fold higher risk for cutaneous melanoma (95% CI: 1.16–1.85) [37]. On the other hand, the meta-analysis of Colantonio et al. reported a higher, but not statistically significant, risk for those younger than 25. The authors mentioned that they included studies using a different cut-off of 20 years as well, to increase the sample size [39].

The development of multiple primary melanomas compared to a single melanoma, was 2.75 times more likely for those with indoor tanning exposure in a case-control melanoma study, even after adjusting for age, a family history of melanoma, the presence of atypical and dysplastic nevi and recreational sun exposure [51].

The melanoma burden attributable to the use of sunbeds is substantial. In the meta-analysis of Wehner et al. (2014), the population proportional attributable risk was 2.6%–9.4% for melanoma, translating to 11,374 melanoma cases each year attributable to indoor tanning in the United States, Europe, and Australia [12]. Based on prevalence data from GLOBOCAN 2008, in Western Europe, an estimated 3438 cases of melanoma could be attributable to sunbed use, most developing among women [18]. The melanoma burden attributable to the use of sunbeds in the French population over 30 years old, was reported to be 4.6% of melanoma cases in women and 1.5% in men in 2015 [52]. In these studies, the melanomas attributed to sunbed use were associated with high intensity of sunbed use (>10 sessions/year) and were more frequently observed in younger individuals [52,53].

A causal relationship between sunbed use and melanoma was proposed in the review of Suppa et al., based on the discussion that the epidemiological criteria of causality [54] are met, including the strength of association, dose response, temporality of the association, consistency, specificity, and plausibility of mechanism and analogy (the effect of the similar factor of sun exposure on melanoma risk is well established) [55]. 

## 5. Sunbed Use and Risk of Keratinocyte Carcinoma (cSCC and BCC)

There are fewer studies on indoor tanning and risk of keratinocyte carcinoma. Published evidence supports that indoor tanning is an independent risk factor for the development of cSCC [56,57,58,59,60]. Furthermore, a dose-dependent association of sunbed use and cSCC development in women has been reported in the prospective large questionnaire studies in the Melanoma in Southern Sweden (MISS) cohort [56] and in the Norwegian Women and Cancer study cohort [57]. The meta-analyses reporting indoor tanning and risk of cSCC are presented in Table 3. The meta-analysis of Wehner et al. (2012), included six studies with patients with cSCC and reported a 67% higher risk for those that had ever exposure to indoor tanning compared to never exposure (RR: 1.67, 95% CI: 1.29–2.17) [40]. Similarly, in the most recent meta-analysis of An et al. (2021), there was a significant risk for cSCC with indoor tanning (RR: 1.58, 95% CI: 1.38–1.81) [37]. This meta-analysis also reported dose–response effects of sunbed use on the risk of cSCC development, while younger age (<20) at first exposure to indoor tanning was not associated with significantly higher risk of cSCC [37] (Table 3).

Regarding BCC, sunbed use was a risk factor for sporadic basal cell carcinoma in Germany [61]. A study from the Icelandic Cancer Registry reported a higher annual percentage change and multiplicity of BCC in women than in men in all age groups [62]. Although sunbed use was not assessed, it was suggested that the parallel widespread use of tanning beds in Iceland, especially among younger women, may have contributed to this sex-specific trend [63]. A US study in the Nurses’ Health Study II (73,494 female nurses with 20-year follow-up) reported a statistically significant higher risk of BCC (HR: 1.15, 95% CI: 1.11–1.19) with the use of tanning beds of four times per year [64]. The meta-analyses reporting indoor tanning and risk of BCC are presented in Table 3. The ever exposure to indoor tanning was associated with a higher risk for BCC in the meta-analysis of Wehner et al. (2012) [40]. However, this was not confirmed as statistically significant in the meta-analysis of An et al. (2021) [37]. The younger age at first exposure to sunbeds has been associated with significantly higher risk of BCC [37,40,64]. Younger age (use during high school/college versus at ages 25–35 years) and more frequent use of tanning beds (more than six times per year compared with no use) were significantly associated with BCC [64]. The meta-analysis of Wehner et al. (2012), showed that exposure to indoor tanning at a younger age was associated with a significantly higher risk for BCC (RR: 1.40, 1.29–1.52) [40], and this was confirmed in the meta-analysis of An et al. (2021) (RR: 1.86, 1.44–2.41) [37].

The KC cases attributable to the use of sunbeds is comparable to the cases of lung cancer attributable to smoking [12]. In the meta-analysis of Wehner et al. (2014), the population proportional attributable risk was 3.0%–10.8% for BCC and 6.7%–21.8% for cSCC, corresponding to 452,796 cases of BCC and SCC each year attributable to indoor tanning in the United States, Europe, and Australia [12].

## 6. Preventive Measures against Sunbed Use

Preventive measures against sunbed use include legislation restricting sunbed use, educational campaigns to inform and discourage from indoor tanning, focusing on high-risk groups, and using the internet, online advertising messages, and social media to reach larger audiences and to promote an untanned appearance.

### 6.1. Restrictive Legislation

In 2009, the World Health Organization classified all forms of sunlamps, tanning beds, and UV light as class 1 carcinogens, which are known to cause cancer in humans [65]. The European Commission, by its Scientific Committee on Health, Environmental and Emerging Risks (SCHEER), stated that ‘based on available evidence, exposure to UVR, including that emitted by sunbeds, causes cutaneous melanoma and SCC at all ages and that the risk for cancer is higher when the first exposure takes place in younger ages’ [66]. In addition, the Association of European cancer Leagues (ECL) has included a message against sunbed use in their European Code Against Cancer (www.cancercode.eu, accessed on 4 November 2022). In particular, within a set of 12 messages to prevent cancer, the UV-related message states: ‘Avoid too much sun, especially for children. Use sun protection. Do not use sunbeds’ [67].

Legislation of indoor tanning restrictions varies across countries. Pawlak et al. provided a comprehensive list of 2011 legislation of indoor tanning restrictions across countries. Indoor tanning laws for youth were implemented, nationwide for eleven countries (France, Spain, Portugal, Germany, Austria, Belgium, England, Wales, Northern Ireland, Scotland, Brazil), as well as in the province of Nova Scotia and the Capital Regional District of British Columbia in Canada, and in 11 states in the United States. In addition, 21 US states require parental consent or accompaniment for tanning bed use [65]. Interestingly, by 2019, three countries had an outright ban (Australia, Brazil, Iran) and 24 countries and 38 states/provinces (in the USA and Canada) had age limits and 15 US states had prohibited unsupervised access for minors. This reflected an increase of 16 countries and 29 states/provinces with age limits since 2011 [13]. The European situation was described more recently in a questionnaire study by Longo et al. in 2019. Within 23 responding countries of the Euromelanoma network, 27% did not report any specific legislation on sunbed use and one-third of the countries did not have a restriction for minors [68]. The inspections related to the use of sunbeds in Denmark, France, Germany, Hungary, Latvia, Norway, Portugal, and United Kingdom reported considerable failure to meet the recommended standards, including enforcement of the age limit and information on potential hazards. Ultraviolet irradiance above the threshold limit (0.3 W/m^2^) was observed in more than 55% of inspected centers, and in more than 90% of centers in some countries. Lack of warning displays and insufficient eye protection was reported in up to 45% centers, and a lack of a mandatory technical inspection in 42% of countries [68]. Furthermore, the European SCHEER recently stated that there is no safe limit for exposure to UV radiation from sunbeds for cosmetic purposes [66].

International regulations on access of minors to sunbeds vary considerably across Australia, Europe and North America (reviewed by Diehl et al., in 2022) [69]. In Australia, there is a total ban on commercial tanning beds, including for minors. In New Zealand, there is a strict ban for minors under the age of 18. In Europe, only 25 out of 47 countries have strict access restrictions for minors. In the UK, a strict ban for minors was reported in England, Northern Ireland, Scotland, and Wales. In the US, there is varied legislation, with a strict ban for minors under the age of 18 in 23 states, and for minors of different age limit in nine states. Twenty-five states have a ‘soft ban’ including access restrictions to minors and the requirements of parental/guardian consent or presence at the tanning salon. In Canada, out of the 12 provinces and territories, 10 have introduced a strict ban for minors under the age of 18 or 19 [69]. A web registry of indoor tanning legislation is provided by the National Conference of State Legislatures in the US. It has been proposed to have a web registry for the continuously evolving indoor tanning legislation around the world, to inform on the current policies across countries and assist towards common advocacy efforts [65].

The implementation of legislation has been reported as an effective strategy to reduce harmful indoor tanning behavior. It was reported that 85% fewer adolescents and 50% fewer adults tanned indoors in the last 12 months in countries that banned indoor tanning for minors (based on studies after 2012) [13] compared with the 2007–2012 period [12]. A systematic review on the impact of US legislation on youth indoor tanning found that indoor tanning prevalence among youth decreased after indoor tanning legislation (3% mean decrease, range:1–6%), especially in states with longer standing indoor tanning legislation (9% mean decrease, range: 2–20%). The small percent differences equate to millions of youths at the population level, suggesting some positive impacts and encouraging results [70]. Additional studies showed that sunbed use in minors has decreased over time since the implementation of a legal ban [71,72,73]. Experience from Australia, where an outright ban of commercial sunbeds has been in place since 2016, showed that this policy intervention was highly effective and had strong public support [74].

In order to inform policy-maker decisions, the health costs and consequences of introducing a ban on commercial indoor tanning have been estimated in England, USA and Europe. In their modeling study, Eden et al. estimated that a nationwide ban on commercial indoor tanning supported by a public health campaign would reduce melanoma and keratinocyte cancers and skin cancer treatment costs in England [75]. Similarly, restricting indoor tanning among minors (<18 years old) in the US would have the potential to reduce melanoma incidence, mortality and save $342.9 million in treatment costs over the lifetime of the 61.2 million youth aged 14 years or younger [76]. Another modeling study by Gordon et al. in 2020 reported that regulatory actions to ban indoor tanning devices could be expected to avert 8.2% (240,000) of melanomas and 7.8% of keratinocyte carcinomas in North America, and to avert 4.9% melanomas and 4.4% keratinocyte carcinomas in Europe [77].

### 6.2. Educational Campaigns

Educational campaigns to inform and discourage from indoor tanning may act in concert with restrictive legislation and reach high-risk groups [13]. The 2019 Euromelanoma study reported the ‘Iberian particularity’ to describe the large difference in the prevalence of sunbed use in Spain (19.3%) and in neighboring Portugal (2%). Interestingly, the authors commented that this difference could be, at least in part, due to the fact that Portugal was part of the Joint Market Surveillance Action on sunbeds and solarium services coordinated by PROSAFE (Product Safety Forum of Europe), that could have raised awareness to avoid the hazards of sunbed use in Portugal [14]. A behavioral strategy to discourage the use of sunbeds could be the display of strong images of skin cancers in indoor tanning facilities [78]. This tactic would follow the use of relevant images put on cigarette packages to discourage smoking, which are more effective in communicating health messages and stimulate cognitive processes than text-only messages [79].

Given the higher risk of skin cancer with an earlier age at first sunbed exposure and the potential to more easily modify behaviors in younger individuals, adolescents are a key target group for educational interventions to inform and shape the choice for untanned skin [80,81,82]. A survey in 31 public high schools from 22 states in the US showed that only 10 high schools (32%) provided curricula regarding sun safety, mainly on the importance of sunscreen, while only one curriculum discussed skin cancer risk. These findings suggest a potential role of school education on sun safety messages, including information on skin cancer risk caused by indoor tanning [83].

Parental influence may also drive the adolescents’ attitude towards indoor tanning, not only by example, but also given the fact that parental consent is necessary for the use of sunbeds by minors in certain states. A US national online survey identified characteristics of parents who were more likely to have positive attitudes towards adolescent indoor tanning: male parents, parent indoor tanning and no reported skin cancer prevention counselling from pediatric healthcare provides. These findings underscore the importance of a family-center approach to disapprove indoor tanning and the role of primary care providers to offer skin cancer prevention counselling [84]. The effects of a social media campaign for mothers (intervention) over 12 months with posts on social media on preventing indoor tanning by adolescent daughters versus control, was assessed in a randomized trial. At six months after the campaign, the intervention-group mothers were less permissive of indoor tanning by daughters, had greater self-efficacy to refuse daughter’s indoor tanning requests, and were more supportive of bans on indoor tanning by minors [85].

Using the internet, online advertising messages, and social media with the aim to reach large, targeted audiences appears a promising and relatively inexpensive tool for public health. Google’s online advertising was used in a pilot study, to deliver targeted prevention messages related to indoor tanning and skin cancer to users entering searches related to tanning beds [86]. Advertisements using wording of educational messages (the truth of tanning beds/the truth beyond UV light/know what tanning beds do) were more popular and had higher click-through rates, compared to messages on health (protect your skin/tanning causes cancer/prevent skin cancer) or appearance (tanning causes winkles/prevent skin aging/tanning makes you ugly) [86]. Google searches for tanning bed key words peak in April and May of each year (before summertime), highlighting the season during which to focus educational and preventive messages [86]. Facebook and Instagram may reach out to the young female audiences that are primary users of indoor tanning [87]. Facebook advertising using three short (<1 min) videos on skin cancer prevention were directed to young women. The videos received 1288 comments and 11,415 reactions. However, there was no significant difference for knowledge between those that were shown the videos compared to those that received their regular newsfeed. There was a short average view duration of the videos (3–9 s) [87].

On the other hand, the marketing and online advertising of sunbed use by the indoor tanning industry is reminiscent of the advertisements of cigarette smoking of the past [88,89]. Social media platforms (e.g., Facebook, Twitter) are used to advertise directly to internet users, promote harmful habits of tanning, and project concerning influences on the beauty and body ideals of children, teenagers, and young adults [90]. The influences of the indoor tanning industry are complex and there is a need to account for industry funding and financial conflict of interests in publications related to indoor tanning. In their systematic review, Adekunle et al., in 2020, reported that 7.2% of articles on indoor tanning had financial links to the indoor tanning industry, and among these, the majority (78%) favored indoor tanning [91].

Clear messages are necessary for raising the public’s awareness on the risks of tanning beds. Educating that tanning is the skin’s defense mechanism trying to compensate for further damage after already being attacked and damaged with UVR exposure may dispel the misinformation that a pre-vacation tan is beneficial. Emphasis on the strict contraindication of indoor tanning with the purpose to increase vitamin D may separate the perceptions of the benefits of vitamin D in normal health that may be acquired by oral vitamin D supplementation from the use of sunbeds that causes skin cancer [92]. The implementation of successful strategies to educate on the risks of indoor tanning and promote an untanned appearance is one part of the equation. The evaluation of the short- and long-term effects of such interventions may select and determine those that are most effective, feasible, and sustainable [89].

## 7. Conclusions

The use of indoor tanning seems to have decreased quite substantially over the years. However, despite the documented harmful effects of tanning and the increasing number of countries adopting restrictive legislation worldwide, there is still a considerable prevalence of sunbed use among adolescents and adults.

Attempting to shed more light on the motivations for tanning-seeking behaviors may pave the way to targeted and impactful strategies to decrease indoor tanning. Clear messages by policy makers, health care systems, mass media campaigns, school curricula, and parents are necessary to raise awareness on the carcinogenicity of indoor UVR tanning and the greater vulnerability of younger people to this carcinogenic impact, and to discourage such exposure. Multicomponent interventions, including educational, mass media, and policy strategies, combined with the use of social media may be more effective towards these ends.

The world has been struggling with an unforeseen viral pandemic. While COVID-19 has put considerable pressure on the health systems and created fear and uncertainty, it might in parallel provide a window of opportunity to put previously cemented beliefs and priorities into question. The preservation of health seems to come more into focus. Through this new lens, the public may come to question the social constructs of beauty produced by magazines, advertisements, social media, and collective opinion. The cost of tanning on health is extremely high to pay.

## Figures and Tables

**Table 1:** A summary of recent publications with international results on the prevalence of indoor UVR tanning across countries.

Reference	Study Type, Study Year	*n*	Prevalence of Ever-Use of Indoor Tanning, by Region	Prevalence of Ever-Use of Indoor Tanning, by Age and Gender (95% CI)	Prevalence of Past-Year Use of Indoor Tanning, by Region	Prevalence of Past-Year Use of Indoor Tanning, by Age and Gender (95% CI)
Wehner, 2014 [12]	Meta-analysis, 1986–2012	491,492	**US** Adults: 35% University students: 59% Adolescents: 17% **Northern and Western Europe** Adults: 42% University students: 12% Adolescents: 24% **Australia** Adults: 11% University students: - Adolescents: 3%	**Adults**: 35.7% (27.5–44.0) Female: 39.8% Male: 20.4% **University students**: 55% (33.0–77.1) Female: 69.3% Male: 40.0% **Adolescents**: 19.3% (14.7–24.0) Female: 31.5% Male: 14.1%	**US** Adults: 13% University students: 43% Adolescents: 10% **Northern and Western Europe** Adults: 21% University students: - Adolescents: 36% **Australia** Adults: 2% University students: - Adolescents: 5%	Adults: 14.0% (11.5–16.5) Female: 19.0% Male: 9.0% **University students**: 43.1% (21.7–64.5) Female: 64.9% Male: 26.8% **Adolescents**: 18.3% (12.6–24.0) Female: 21.3% Male: 7.5%
Rodriguez-Acevedo, 2020 [13]	Meta-analysis, 2009–2018	401,999	**US/Canada** Adults: 35.6% Adolescents: 6.7% **Europe** Adults: 31.3% Adolescents: 16.1% **Australia** Adults: 10.8% Adolescents: -	**Adults**: 31.9% (25.3–39.1) Female: 38.0% Male: 24.9% **Adolescents**: 9.7% (7.3–12.4) Female: 8.4% Male: 4.0%	**US/Canada** Adults: 14.4% Adolescents: 7.6% **Europe** Adults: 11.1% Adolescents: 5.1% **Australia** Adults: 2.5% Adolescents: -	**Adults**: 12.5% (9.5–15.6) Female: 16.8% Male: 8.5% **Adolescents**: 6.7% (4.4–9.6) Female: 8.9% Male: 3.9%
Suppa, 2019 [14]	Survey, 2009–2014	227,888	**30 European countries overall prevalence**: 10.6% **Prevalence: 18–27%** Belgium, Denmark, Estonia, Hungary, Italy, Latvia, Norway, Spain **Prevalence: 10–18%** Croatia, Czech Republic, Germany, Ireland, Lithuania, Poland Russia, Serbia, Sweden **Prevalence: <10%** Bosnia-Herzegovina, Cyprus, Georgia, Greece, Macdonia(FYROM), Malta, Moldova, Portugal, Romania, Slovenia, Switzerland, Turkey, Ukraine	Adults/elderly: 8.3% Young adults: 17.0% Adolescents: 5.9%		

*n*: number of overall participants, UVR: ultraviolet radiation, 95% CI: 95% confidence interval, (-): not reported data.

**Table 2:** Meta-analyses reporting indoor UVR tanning and risk of cutaneous melanoma. Available results by age group and dose/frequency of sunbed use are shown.

Reference (Years of Included Studies)	*n* Studies ^a^	*n* Cases Overall	CM Group Studied	Ever Indoor Tanning (vs. Never) RR (95% CI)	Annual Frequency of Indoor Tanning (Times) RR (95% CI)	Early Age at First Use of Indoor Tanning (Years) RR (95% CI)
An, 2021 [37] (Up to 2021)	36	14,583	**CM**	**1.27 (1.16–1.39)**	<10: 1.33 (1.00–1.78) **≥10: 1.52 (1.22–1.89)**	**<20: 1.47 (1.16–1.85)** **≥20: 1.28 (1.01–1.63)**
	2	1771	**CM < 50 y**	**1.75 (1.14–2.69)**	-	-
Burgard, 2018 [38] (1981–2015)	31	11,706	**CM**	**1.19 (1.05–1.34)**	≤10: 1.13 (0.93–1.38) **>10: 1.43 (1.17–1.74)**	**<25: 1.59 (1.38–1.83)**
Colantonio, 2014 [39] (1981–2012)	31	14,956	**CM**	**1.16 (1.05–1.28)**	≤10:1.07 (0.90–1.26) **>10: 1.34 (1.05–1.71)**	<25: 1.35 (0.99–1.84) ≥25: 1.11 (0.86–1.42)
Boniol, 2012 [18] (1981–2012)	27	11,428	**CM**	**1.20 (1.08–1.34)**	**High:1.42 (1.15–1.74)**	**<35: 1.59 (1.36–1.85) ^b^**
IARC, 2007 [41] (1979–2005)	19	7355	CM	1.15 (1.00–1.31)	-	**<35: 1.75 (1.35–2.26)**
Gallagher, 2005 [42] (1984–2004)	10	4560	**CM**	**1.25 (1.05–1.49)**	**Longest: 1.61 (1.21–2.12) ^c^**	**Young adult: 1.69 (1.32–2.18) ^d^**

UVR: ultraviolet radiation, *n*: number, CM: cutaneous melanoma, y: years old, RR: relative risk, 95% CI: 95% confidence interval. (-): not reported data. Statistically significant results are shown in bold. ^a^ Different studies were included in different meta-analyses, accounting for the differences in the number of overall included cases. Apart from adding new studies, the most recent meta-analysis of An et al., 2021, did not include 8 studies that were included in the previous meta-analysis by Colantonio et al., 2014 (Beitner 1990, Gallagher 1986, Dubin 1989, Holly 1987, Kaskel 2001, Klepp and Magnus 1979, Rodenas 1996, Schmitt 2011). The meta-analysis of Boniol et al., updated the meta-analysis by IARC. ^b^ correction by Boniol et al. [43]. ^c^ Included longest duration or highest frequency of use. ^d^ Included studies with different age cut-offs ranging from 10–19 years to <30.

**Table 3:** Meta-analyses reporting indoor UVR tanning and risk of keratinocyte carcinoma. Available results by age group and dose/frequency of sunbed use are shown.

Reference (Years of Included Studies)	*n* Studies	*n* Cases Overall	KC Studied	Ever Indoor Tanning (vs. Never) RR (95% CI)	Annual Frequency of Indoor Tanning (Times) RR (95% CI)	Early Age at First Use of Indoor Tanning (Years) RR (95% CI)
An, 2021 [37] (Up to 2021)	18	10,406	**KC**	**1.40 (1.18–1.65)**	**<10: 1.32 (1.14–1.52)** **≥10: 1.56 (1.31–1.86)**	**<20: 2.02 (1.44–2.83)** **≥20: 1.48 (1.31–1.68)**
	4	1410	**KC < 50 y**	**1.81 (1.38–2.37)**	-	-
	9	2528	**cSCC**	**1.58 (1.38–1.81)**	**<10: 1.46 (1.24–1.71)** **≥10: 1.65 (1.30–2.10)**	<20: 1.89 (0.90–3.98) **≥20: 1.53 (1.26–1.85)**
	1	131	**cSCC < 50 y**	**1.99 (1.48–2.68)**	-	-
	10	7643	BCC	1.24 (1.00–1.55)	**<10: 1.29 (1.01–1.65)** **≥10: 1.46 (1.28–1.66)**	**<20: 1.86 (1.44–2.41)** **≥20: 1.51 (1.19–1.92)**
	3	1049	**BCC < 50 y**	**1.79 (1.15–2.77)**	-	-
Boniol, 2012 [18] (1981–2012)	5	1242	**cSCC**	**2.23 (1.39–3.570)**	-	-
	6	6995	**BCC**	**1.09 (1.01–1.18)**	-	-
Wehner, 2012 [40] (1985–2012)	12	9328	KC	-	-	-
	6	-	**cSCC**	**1.67 (1.29–2.17)**	-	Young: 2.02 (0.70–5.86)
	8	-	**BCC**	**1.29 (1.08–1.53)**	High: 1.50 (0.81–2.77)	**Young: 1.40 (1.29–1.52)**
IARC, 2007 [41] (1979–2005)	3	-	**cSCC**	**2.25 (1.08–4.70)**	-	-
	4	-	BCC	1.03 (0.56–1.90)	-	-

UVR: ultraviolet radiation, *n*: number, KC: keratinocyte carcinoma consisting of cSCC and BCC, also termed as nonmelanoma skin cancer), cSCC: cutaneous squamous cell carcinoma, BCC: basal cell carcinoma, y: years old. RR: relative risk, 95% CI: 95% confidence interval. (-): not reported data. Statistically significant results are shown in bold.

## References

[1] Calzavara-Pinton P.G., Arisi M., Wolf P. (2019). Sunbeds and carcinogenesis: The need for new regulations and restrictions in Europe from the Euromelanoma perspective. J. Eur. Acad. Derm. Venereol..

[2] Dessinioti C., Stratigos A.J., Rigopoulos D., Katsambas A.D. (2009). A review of genetic disorders of hypopigmentation: Lessons learned from the biology of melanocytes. Exp. Derm..

[3] Lin J.Y., Fisher D.E. (2007). Melanocyte biology and skin pigmentation. Nature.

[4] Flanagan N., Healy E., Ray A., Philips S., Todd C., Jackson I.J., Birch-Machin M.A., Rees J.L. (2000). Pleiotropic effects of the melanocortin 1 receptor (MC1R) gene on human pigmentation. Hum. Mol. Genet..

[5] Dessinioti C., Antoniou C., Katsambas A., Stratigos A.J. (2011). Melanocortin 1 receptor variants: Functional role and pigmentary associations. Photochem. Photobiol..

[6] Dessinioti C., Antoniou C., Katsambas A., Stratigos A.J. (2010). Basal cell carcinoma: What’s new under the sun. Photochem. Photobiol..

[7] Kripke M.L., Cox P.A., Alas L.G., Yarosh D.B. (1992). Pyrimidine dimers in DNA initiate systemic immunosuppression in UV-irradiated mice. Proc. Natl. Acad. Sci. USA.

[8] Walterscheid J.P., Ullrich S.E., Nghiem D.X. (2002). Platelet-activating factor, a molecular sensor for cellular damage, activates systemic immune suppression. J. Exp. Med..

[9] Noonan F.P., De Fabo E.C. (1992). Immunosuppression by ultraviolet B radiation: Initiation by urocanic acid. Immunol. Today.

[10] Nilsen L.T., Hannevik M., Veierod M.B. (2016). Ultraviolet exposure from indoor tanning devices: A systematic review. Br. J. Derm..

[11] IARC Working Group on the Evluation of Carcinogenic Risks to Humans (2012). A review of human carcinogens. Part D: Radiation. IARC Monographs on the Evaluation of Carcinogenic Risks to Humans Series.

[12] Wehner M.R., Chren M.M., Nameth D., Choudhry A., Gaskins M., Nead K.T., Boscardin W.J., Linos E. (2014). International prevalence of indoor tanning: A systematic review and meta-analysis. JAMA Derm..

[13] Rodriguez-Acevedo A.J., Green A.C., Sinclair C., van Deventer E., Gordon L.G. (2020). Indoor tanning prevalence after the International Agency for Research on Cancer statement on carcinogenicity of artificial tanning devices: Systematic review and meta-analysis. Br. J. Derm..

[14] Suppa M., Gandini S., Njimi H., Bulliard J.L., Correia O., Duarte A.F., Peris K., Stratigos A.J., Nagore E., Longo M.I. (2019). Prevalence and determinants of sunbed use in thirty European countries: Data from the Euromelanoma skin cancer prevention campaign. J. Eur. Acad. Derm. Venereol..

[15] Suppa M., Gandini S., Bulliard J.L., Daxhelet M., Zamagni M., Forsea A.M., Longo M.I., Del Marmol V. (2019). Who, why, where: An overview of determinants of sunbed use in Europe. J. Eur. Acad. Derm. Venereol..

[16] Mastroeni S., Sampogna F., Salcedo N.M., Ricci F., Fania L., Antonelli F., Abeni D., Cristofolini M. (2021). Factors associated with sunbed use among 3692 outpatients in 18 centers of the Italian Cancer League (LILT). Sci. Rep..

[17] Bataille V., Boniol M., De Vries E., Severi G., Brandberg Y., Sasieni P., Cuzick J., Eggermont A., Ringborg U., Grivegnee A.R. (2005). A multicentre epidemiological study on sunbed use and cutaneous melanoma in Europe. Eur. J. Cancer.

[18] Boniol M., Autier P., Boyle P., Gandini S. (2012). Cutaneous melanoma attributable to sunbed use: Systematic review and meta-analysis. BMJ.

[19] Bentzen J., Krarup A.F., Castberg I.M., Jensen P.D., Philip A. (2013). Determinants of sunbed use in a population of Danish adolescents. Eur. J. Cancer Prev..

[20] Koster B., Thorgaard C., Clemmensen I.H., Philip A. (2009). Sunbed use in the Danish population in 2007: A cross-sectional study. Prev. Med..

[21] Krarup A.F., Koster B., Thorgaard C., Philip A., Clemmensen I.H. (2011). Sunbed use by children aged 8-18 years in Denmark in 2008: A cross-sectional study. Br. J. Derm..

[22] Boldeman C., Beitner H., Jansson B., Nilsson B., Ullen H. (1996). Sunbed use in relation to phenotype, erythema, sunscreen use and skin diseases. A questionnaire survey among Swedish adolescents. Br. J. Derm..

[23] Boldeman C., Jansson B., Nilsson B., Ullen H. (1997). Sunbed use in Swedish urban adolescents related to behavioral characteristics. Prev. Med..

[24] Boldeman C., Branstrom R., Dal H., Kristjansson S., Rodvall Y., Jansson B., Ullen H. (2001). Tanning habits and sunburn in a Swedish population age 13–50 years. Eur. J. Cancer.

[25] Moan J.E., Baturaite Z., Grigalavicius M., Juzeniene A. (2013). Sunbed use and cutaneous melanoma in Norway. Scand J. Public Health.

[26] Coups E.J., Phillips L.A. (2011). A more systematic review of correlates of indoor tanning. J. Eur. Acad. Derm. Venereol..

[27] Schneider S., Kramer H. (2010). Who uses sunbeds? A systematic literature review of risk groups in developed countries. J. Eur. Acad. Derm. Venereol..

[28] Mansh M., Katz K.A., Linos E., Chren M.M., Arron S. (2015). Association of Skin Cancer and Indoor Tanning in Sexual Minority Men and Women. JAMA Derm..

[29] Singer S., Tkachenko E., Yeung H., Mostaghimi A. (2020). Skin cancer and skin cancer risk behaviors among sexual and gender minority populations: A systematic review. J. Am. Acad. Derm..

[30] Admassu N., Pimentel M.A., Halley M.C., Torres J., Pascua N., Katz K.A., Linos E. (2019). Motivations among sexual-minority men for starting and stopping indoor tanning. Br. J. Derm..

[31] Wester U., Boldemann C., Jansson B., Ullen H. (1999). Population UV-dose and skin area--do sunbeds rival the sun?. Health Phys..

[32] Bali R., Ji-Xu A., Felton S.J. (2022). The significant health threat from sunbed use as a self treatment in patients with acne. Clin. Exp. Dermatol..

[33] Nast A., Dreno B., Bettoli V., Bukvic Mokos Z., Degitz K., Dressler C., Finlay A.Y., Haedersdal M., Lambert J., Layton A. (2016). European evidence-based (S3) guideline for the treatment of acne-update 2016-short version. J. Eur. Acad. Derm. Venereol..

[34] Pagoto S.L., Nahar V.K., Frisard C., Conroy D.E., Lemon S.C., Oleski J., Hillhouse J. (2018). A Comparison of Tanning Habits Among Gym Tanners and Other Tanners. JAMA Derm..

[35] Diehl K., Breitbart E.W., de Buhr Y., Schneider S., Gorig T. (2022). Shift in place of tanning bed use from tanning salons to spa, fitness, and beauty facilities: A trend perspective. Photodermatol. Photoimmunol. Photomed..

[36] Huang C.M., Kirchhof M.G. (2017). A Cross-Sectional Study of Indoor Tanning in Fitness Centres. J. Cutan Med. Surg..

[37] An S., Kim K., Moon S., Ko K.P., Kim I., Lee J.E., Park S.K. (2021). Indoor Tanning and the Risk of Overall and Early-Onset Melanoma and Non-Melanoma Skin Cancer: Systematic Review and Meta-Analysis. Cancers.

[38] Burgard B., Schope J., Holzschuh I., Schiekofer C., Reichrath S., Stefan W., Pilz S., Ordonez-Mena J., Marz W., Vogt T. (2018). Solarium Use and Risk for Malignant Melanoma: Meta-analysis and Evidence-based Medicine Systematic Review. Anticancer Res..

[39] Colantonio S., Bracken M.B., Beecker J. (2014). The association of indoor tanning and melanoma in adults: Systematic review and meta-analysis. J. Am. Acad. Derm..

[40] Wehner M.R., Shive M.L., Chren M.M., Han J., Qureshi A.A., Linos E. (2012). Indoor tanning and non-melanoma skin cancer: Systematic review and meta-analysis. BMJ.

[41] International Agency for Research on Cancer Working Group on Artificial Ultraviolet Light and Skin Cancer (2007). The association of use of sunbeds with cutaneous malignant melanoma and other skin cancers: A systematic review. Int. J. Cancer.

[42] Gallagher R.P., Spinelli J.J., Lee T.K. (2005). Tanning beds, sunlamps, and risk of cutaneous malignant melanoma. Cancer Epidemiol. Biomark. Prev..

[43] Boniol M., Autier P., Boyle P., Gandini S. (2012). Correction: Cutaneous melanoma attributable to sunbed use: Systematic review and meta-analysis. BMJ.

[44] Lazovich D., Isaksson Vogel R., Weinstock M.A., Nelson H.H., Ahmed R.L., Berwick M. (2016). Association Between Indoor Tanning and Melanoma in Younger Men and Women. JAMA Derm..

[45] Ghiasvand R., Rueegg C.S., Weiderpass E., Green A.C., Lund E., Veierod M.B. (2017). Indoor Tanning and Melanoma Risk: Long-Term Evidence From a Prospective Population-Based Cohort Study. Am. J. Epidemiol..

[46] Cust A.E., Armstrong B.K., Goumas C., Jenkins M.A., Schmid H., Hopper J.L., Kefford R.F., Giles G.G., Aitken J.F., Mann G.J. (2011). Sunbed use during adolescence and early adulthood is associated with increased risk of early-onset melanoma. Int. J. Cancer.

[47] Veierod M.B., Weiderpass E., Thorn M., Hansson J., Lund E., Armstrong B., Adami H.O. (2003). A prospective study of pigmentation, sun exposure, and risk of cutaneous malignant melanoma in women. J. Natl. Cancer Inst..

[48] Nielsen K., Masback A., Olsson H., Ingvar C. (2012). A prospective, population-based study of 40,000 women regarding host factors, UV exposure and sunbed use in relation to risk and anatomic site of cutaneous melanoma. Int. J. Cancer.

[49] Gandini S., Dore J.F., Autier P., Greinert R., Boniol M. (2019). Epidemiological evidence of carcinogenicity of sunbed use and of efficacy of preventive measures. J. Eur. Acad. Derm. Venereol..

[50] Veierod M.B., Adami H.O., Lund E., Armstrong B.K., Weiderpass E. (2010). Sun and solarium exposure and melanoma risk: Effects of age, pigmentary characteristics, and nevi. Cancer Epidemiol. Biomark. Prev..

[51] Karapetyan L., Yang X., Wang H., Sander C.A., Moyer A., Wilson M., Karunamurthy A., Kirkwood J.M. (2021). Indoor tanning exposure in association with multiple primary melanoma. Cancer.

[52] Arnold M., Kvaskoff M., Thuret A., Guenel P., Bray F., Soerjomataram I. (2018). Cutaneous melanoma in France in 2015 attributable to solar ultraviolet radiation and the use of sunbeds. J. Eur. Acad. Derm. Venereol..

[53] Gredner T., Behrens G., Stock C., Brenner H., Mons U. (2018). Cancers Due to Infection and Selected Environmental Factors. Dtsch. Arztebl. Int..

[54] Hill A.B. (1965). The Environment and Disease: Association or Causation?. Proc. R Soc. Med..

[55] Suppa M., Gandini S. (2019). Sunbeds and melanoma risk: Time to close the debate. Curr. Opin. Oncol..

[56] Christensen G.B., Ingvar C., Hartman L.W., Olsson H., Nielsen K. (2019). Sunbed Use Increases Cutaneous Squamous Cell Carcinoma Risk in Women: A Large-scale, Prospective Study in Sweden. Acta. Derm Venereol..

[57] Lergenmuller S., Ghiasvand R., Robsahm T.E., Green A.C., Lund E., Rueegg C.S., Veierod M.B. (2019). Association of Lifetime Indoor Tanning and Subsequent Risk of Cutaneous Squamous Cell Carcinoma. JAMA Derm..

[58] Liu F.C., Grimsrud T.K., Veierod M.B., Robsahm T.E., Ghiasvand R., Babigumira R., Shala N.K., Stenehjem J.S. (2021). Ultraviolet radiation and risk of cutaneous melanoma and squamous cell carcinoma in males and females in the Norwegian Offshore Petroleum Workers cohort. Am. J. Ind. Med..

[59] Veierod M.B., Couto E., Lund E., Adami H.O., Weiderpass E. (2014). Host characteristics, sun exposure, indoor tanning and risk of squamous cell carcinoma of the skin. Int. J. Cancer.

[60] Tierney P., de Gruijl F.R., Ibbotson S., Moseley H. (2015). Predicted increased risk of squamous cell carcinoma induction associated with sunbed exposure habits. Br. J. Derm..

[61] Kaskel P., Lange U., Sander S., Huber M.A., Utikal J., Leiter U., Krahn G., Meurer M., Kron M. (2015). Ultraviolet exposure and risk of melanoma and basal cell carcinoma in Ulm and Dresden, Germany. J. Eur. Acad. Derm. Venereol..

[62] Adalsteinsson J.A., Ratner D., Olafsdottir E., Grant-Kels J., Ungar J., Silverberg J.I., Kristjansson A.K., Jonasson J.G., Tryggvadottir L. (2020). Basal cell carcinoma: An emerging epidemic in women in Iceland. Br. J. Derm..

[63] Pandeya N. (2020). Rising incidence of basal cell carcinoma in women in Iceland: Is it sunbed use?. Br. J. Derm..

[64] Zhang M., Qureshi A.A., Geller A.C., Frazier L., Hunter D.J., Han J. (2012). Use of tanning beds and incidence of skin cancer. J. Clin. Oncol..

[65] Pawlak M.T., Bui M., Amir M., Burkhardt D.L., Chen A.K., Dellavalle R.P. (2012). Legislation restricting access to indoor tanning throughout the world. Arch. Derm..

[66] SCHEER (Scientific Committee on Health EaER) Opinion on biological effects of ultraviolet radiation relevant to health with particular reference to sunbeds for cosmetic purposes. https://health.ec.europa.eu/other-pages/health-sc-basic-page/opinion-biological-effects-ultraviolet-radiation-relevant-health-particular-reference-sunbeds_en#modal.

[67] Yared W., Boonen B., McElwee G., Ferguson M. (2019). Cancer league actions against sunbed use for skin cancer prevention. J. Eur. Acad. Derm. Venereol..

[68] Longo M.I., Bulliard J.L., Correia O., Maier H., Magnusson S.M., Konno P., Goad N., Duarte A.F., Olah J., Nilsen L.T.N. (2019). Sunbed use legislation in Europe: Assessment of current status. J. Eur. Acad. Derm. Venereol..

[69] Diehl K., Lindwedel K.S., Mathes S., Gorig T., Gefeller O. (2022). Tanning Bed Legislation for Minors: A Comprehensive International Comparison. Children.

[70] Reimann J., McWhirter J.E., Cimino A., Papadopoulos A., Dewey C. (2019). Impact of legislation on youth indoor tanning behaviour: A systematic review. Prev. Med..

[71] Gordon L.G., Hainsworth R., Eden M., Epton T., Lorigan P., Grant M., Green A.C., Payne K. (2021). Sunbed Use among 11- to 17-Year-Olds and Estimated Number of Commercial Sunbeds in England with Implications for a ‘Buy-Back’ Scheme. Children.

[72] Qin J., Holman D.M., Jones S.E., Berkowitz Z., Guy G.P. (2018). State Indoor Tanning Laws and Prevalence of Indoor Tanning Among US High School Students, 2009–2015. Am. J. Public Health.

[73] Diehl K., Gorig T., Greinert R., Breitbart E.W., Schneider S. (2019). Trends in Tanning Bed Use, Motivation, and Risk Awareness in Germany: Findings from Four Waves of the National Cancer Aid Monitoring (NCAM). Int. J. Env. Res. Public Health.

[74] Janda M., Sinclair C. (2022). Experience from an outright ban of commercial sunbeds in the Australian context. Br. J. Derm..

[75] Eden M., Hainsworth R., Gordon L.G., Epton T., Lorigan P., Rhodes L.E., Marais R., Green A.C., Payne K. (2022). Cost-effectiveness of a policy-based intervention to reduce melanoma and other skin cancers associated with indoor tanning. Br. J. Derm..

[76] Guy G.P., Zhang Y., Ekwueme D.U., Rim S.H., Watson M. (2017). The potential impact of reducing indoor tanning on melanoma prevention and treatment costs in the United States: An economic analysis. J. Am. Acad. Derm..

[77] Gordon L.G., Rodriguez-Acevedo A.J., Koster B., Guy G.P., Sinclair C., Van Deventer E., Green A.C. (2020). Association of Indoor Tanning Regulations With Health and Economic Outcomes in North America and Europe. JAMA Derm..

[78] Sontag J.M., Noar S.M. (2017). Assessing the Potential Effectiveness of Pictorial Messages to Deter Young Women from Indoor Tanning: An Experimental Study. J. Health Commun..

[79] Hammond D. (2011). Health warning messages on tobacco products: A review. Tob. Control..

[80] Boyle R., O’Hagan A.H., Donnelly D., Donnelly C., Gordon S., McElwee G., Gavin A. (2010). Trends in reported sun bed use, sunburn, and sun care knowledge and attitudes in a U.K. region: Results of a survey of the Northern Ireland population. Br. J. Derm..

[81] Koster B., Thorgaard C., Philip A., Clemmensen H. (2011). Sunbed use and campaign initiatives in the Danish population, 2007–2009: A cross-sectional study. J. Eur. Acad. Derm. Venereol..

[82] Aarestrup C., Bonnesen C.T., Thygesen L.C., Krarup A.F., Waagstein A.B., Jensen P.D., Bentzen J. (2014). The effect of a school-based intervention on sunbed use in Danish pupils at continuation schools: A cluster-randomized controlled trial. J. Adolesc. Health.

[83] Tripathi R., Tamashunas N.L., Xiang L., Simmons E., Mazmudar R.S., Bordeaux J.S., Scott J.F. (2022). Limited sun safety education in high school curricula: A pilot study and call to action. Arch. Dermatol. Res..

[84] Feng J., Kim Y., Kornides M.L., McRee A.L., Mays D., Asgari M.M., Gilkey M.B. (2018). Correlates of positive parental attitudes towards adolescent indoor tanning in the USA. Br. J. Derm..

[85] Buller D.B., Pagoto S., Henry K.L., Baker K., Walkosz B.J., Hillhouse J., Berteletti J., Bibeau J., Kinsey A. (2022). Persisting Effects of a Social Media Campaign to Prevent Indoor Tanning: A Randomized Trial. Cancer Epidemiol. Biomark. Prev..

[86] Cidre Serrano W., Chren M.M., Resneck J.S., Aji N.N., Pagoto S., Linos E. (2016). Online Advertising for Cancer Prevention: Google Ads and Tanning Beds. JAMA Derm..

[87] Morrison L., Chen C., Torres J.S., Wehner M., Junn A., Linos E. (2019). Facebook advertising for cancer prevention: A pilot study. Br. J. Derm..

[88] Stapleton J.L., Darabos K., Carpenter A., Lewis M.J., Greene K., Hudson S.V. (2015). Indoor tanning users’ experiences with tanning salon direct to consumer marketing. J. Am. Acad. Derm..

[89] Holman D.M., Fox K.A., Glenn J.D., Guy G.P., Watson M., Baker K., Cokkinides V., Gottlieb M., Lazovich D., Perna F.M. (2013). Strategies to reduce indoor tanning: Current research gaps and future opportunities for prevention. Am. J. Prev. Med..

[90] Falzone A.E., Brindis C.D., Chren M.M., Junn A., Pagoto S., Wehner M., Linos E. (2017). Teens, Tweets, and Tanning Beds: Rethinking the Use of Social Media for Skin Cancer Prevention. Am. J. Prev. Med..

[91] Adekunle L., Chen R., Morrison L., Halley M., Eng V., Hendlin Y., Wehner M.R., Chren M.M., Linos E. (2020). Association between financial links to indoor tanning industry and conclusions of published studies on indoor tanning: Systematic review. BMJ.

[92] Pierret L., Suppa M., Gandini S., Del Marmol V., Gutermuth J. (2019). Overview on vitamin D and sunbed use. J. Eur. Acad. Derm. Venereol..

